# Supplementary material for: The importance of forgetting: Limiting memory improves recovery of topological characteristics from neural data
Source: PLoS One. 2018 Sep 4;13(9):e0202561. doi: 10.1371/journal.pone.0202561 (PMC6122934; doi:10.1371/journal.pone.0202561)
Supplement: S1 File — (PDF) [file pone.0202561.s001.pdf]

SUPPLEMENTARY INFORMATION TO ACCOMPANY "THE IMPORTANCE OF FORGETTING: LIMITING MEMORY IMPROVES RECOVERY OF TOPOLOGICAL CHARACTERISTICS FROM NEURAL DATA".

SAMIR CHOWDHURY, FACUNDO MÉMOLI, BOWEN DAI

Persistent homology

A good reference for the concepts in this section is [1].

Given a finite set X, a *simplicial complex* is a collection K_X of nonempty subsets of X such that whenever $\sigma \in K_X$, any subset $\tau \subseteq \sigma$ also belongs to K_X. The singleton elements in this collection are the *vertices* of the simplicial complex, the two-element subsets of X belonging to this collection are the *edges*, and for any $k \in \mathbb{Z}_+$, the $(k+1)$ element subsets of X in this collection are the k-simplices.

Simplicial complexes are useful tools for constructing topological models of discrete data. One can systematically construct simplicial complexes at various resolutions and then study these topological models to understand phenomena in the original dataset at various levels of granularity.

Given a nested sequence of simplicial complexes $K_1 \subseteq K_2 \subseteq K_3 \subseteq \ldots$, one can perform an operation referred to as *taking homology with field coefficients* to obtain a sequence of finite-dimensional vector spaces with linear maps $H_d(K_1) \to H_d(K_2) \to H_d(K_3) \to \ldots$. The subscript d can be any number $0, 1, 2, 3, \ldots$, and each choice of d leads to a different interpretation of the rank of $H_d(K_\bullet)$. Assuming that the simplicial complexes are constructed from some initial dataset, these interpretations include the number of clusters in the data ($d = 0$), the number of loops in the data ($d = 1$), and so on.

The sequence of vector spaces with linear maps above is called a *persistent vector space*. The rank information contained in such an object has a convenient visual representation as a *persistence barcode*, i.e. as a set of lines over a single axis. A 0-dimensional barcode tracks the resolutions at which clusters in data merge together, a 1-dimensional barcode tracks the appearance (and disappearance) of loops in data, and so on.

Zigzag persistence

We refer the reader to [2, 3] for additional details on zigzag persistence.

In standard persistent homology, one of the restrictions on the sequence of simplicial complexes $K_1 \subseteq K_2 \subseteq K_3 \subseteq \ldots$ is that the complexes are only allowed to grow, not reduce. *Zigzag* persistent homology [3] is a generalization of standard persistent homology that overcomes this limitation. In this setting, we have a collection of simplicial complexes $K_1 \leftrightarrow K_2 \leftrightarrow \ldots \leftrightarrow K_n$ where each $\leftrightarrow$ represents either an inclusion $K_{n-1} \subseteq K_n$, or an inclusion $K_{n-1} \supseteq K_n$. Such a sequence is called a *zigzag filtration*. Notice that a zigzag filtration models a dynamic simplicial complex, where simplices are sequentially added or deleted. In particular, the synaptic potentiation complex is an example of a zigzag filtration.

The theory of zigzag persistent homology guarantees that applying homology with field coefficients (in dimension d) returns a sequence of vector spaces $\{H_d(K_i)\}_{i=1}^{n}$ with linear maps in the directions of the corresponding inclusions. A persistence barcode may then be plotted as before.

All our zigzag persistence computations were carried out using `Dionysus 1` [4].

References

[1] Herbert Edelsbrunner and John Harer. *Computational topology: an introduction*. American Mathematical Soc., 2010.

[2] Gunnar Carlsson, Vin De Silva, and Dmitriy Morozov. Zigzag persistent homology and real-valued functions. In *Proceedings of the twenty-fifth annual symposium on Computational geometry*, pages 247–256. ACM, 2009.

[3] Gunnar Carlsson and Vin De Silva. Zigzag persistence. *Foundations of computational mathematics*, 10(4):367–405, 2010.

[4] Dmitriy Morozov. Dionysus. *Software available at http://www. mrzv. org/software/dionysus*, 2012.
